# Genetic Analysis of Fruit Quality Traits in Sweet Watermelon (*Citrullus lanatus* var. *lanatus*): A Review

**DOI:** 10.3389/fpls.2022.834696

**Published:** 2022-03-22

**Authors:** Jacob Mashilo, Hussein Shimelis, Richard Mantlo Ngwepe, Zamalotshwa Thungo

**Affiliations:** ^1^Limpopo Department of Agriculture and Rural Development, Agriculture Regulatory and Technology Development Directorate, Crop Science Division, Towoomba Research Station, Bela-Bela, South Africa; ^2^African Centre for Crop Improvement, University of KwaZulu-Natal, Pietermaritzburg, South Africa; ^3^Vegetable, Industrial and Medicinal Plants, Agricultural Research Council, Pretoria, South Africa

**Keywords:** *C. lanatus*, flesh texture, gene editing, molecular markers, phytochemical compounds, phytohormones, transcriptome analysis, quantitative trait loci

## Abstract

Fruit quality traits of sweet watermelon (*Citrullus lanatus* var. *lanatus*) are crucial for new product development and commercialization. Sweet watermelon fruit quality traits are affected by the compositions of phytochemical compounds, phytohormones, and fruit flesh firmness which are affected by genes, the growing environment and their interaction. These compositions determine fruit ripening, eating quality, and postharvest shelf-life. Knowledge of the genetic profile and analyses of quality traits in watermelon is vital to develop improved cultivars with enhanced nutritional compositions, consumer-preferred traits, and extended storage life. This review aims to present the opportunities and progress made on the genetic analysis of fruit quality traits in watermelon as a guide for quality breeding based on economic and end-user attributes. The first section of the review highlights the genetic mechanisms involved in the biosynthesis of phytochemical compounds (i.e., sugars, carotenoids, amino acids, organic acids, and volatile compounds), phytohormones (i.e., ethylene and abscisic acid) and fruit flesh structural components (i.e., cellulose, hemicellulose, and pectin) elicited during watermelon fruit development and ripening. The second section pinpoints the progress on the development of molecular markers and quantitative trait loci (QTL) analysis for phytochemical compounds, phytohormones and fruit quality attributes. The review presents gene-editing technology and innovations associated with fruit quality traits for selection and accelerated cultivar development. Finally, the paper discussed gene actions conditioning fruit ripening in citron watermelon (*C. lanatus var. citroides* [L. H. Bailey] Mansf. ex Greb.) as reference genetic resources to guide current and future breeding. Information presented in this review is useful for watermelon variety design, product profiling and development to serve the diverse value chains of the crop.

## Graphical Abstract

An overview of phytochemical compounds, phytohormones and flesh textural properties influencing fruit quality of watermelon fruit, and developed genomic and genetic resources for fruit quality breeding.

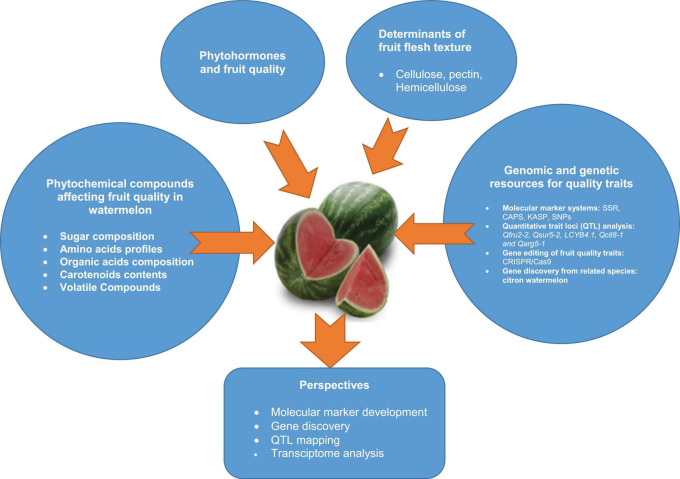

## Introduction

Sweet watermelon [*Citrullus lanatus* (Thunb.) Matsum. and Nakai var. *lanatus*; 2n = 2x = 22] belonging to the family Cucurbitaceae is predominantly grown for its fresh and nutritious fruit. The genus *Citrullus* comprises several species: *C. lanatus* var. *citroides, C. mucosospermus*, *C. colocynthis*, *C. ecirrhosus*, *C. rehmii*, and *C. naudinianus* (Chomicki and Renner, 2015). The center of origin and genetic diversity of most *Citrullus* species is continental Africa (Robinson and Decker-Walters, 1997; Rubatzky, 2001; Dane and Lang, 2004).

With an estimated total annual production of ∼90 million tons, watermelon ranks in the top 10 fruit crops widely produced globally (FAOSTAT, 2020). China is the largest producer of watermelons, accounting for 60 million tons per annum from an estimated agricultural land of 1.4 million ha (FAOSTAT, 2020). The following leading global producers of watermelons are Turkey and Iran, with an estimated annual total production of 3.4 and 2.7 million tons, respectively. Brazil and Egypt rank fourth and fifth with total production of 2.1 and 1.4 million tons per annum, respectively. The United States ranks sixth with 1.74 million tons total production annually. The economic and nutritional values of watermelon have been recognized in recent years, creating the opportunity to develop and commercialize new varieties combining high fruit yield and quality (Yang et al., 2016).

Watermelon fruit is a rich source of water and human health-benefiting nutrients. The fruit consists of sugars (i.e., fructose, sucrose, and glucose), carotenoids (i.e., lycopene and β-carotene), amino acids (i.e., citrulline and arginine), organic acids (i.e., citric and glutamic acids) and volatile compounds (VOCs) (Perkins-Veazie et al., 2006; Davis et al., 2011; Liu et al., 2012; Yoo et al., 2012; Ren et al., 2014; Jawad et al., 2020). The fruit sugar content determines the sweetness, while the VOCs influence aroma and flavor, and the organic acids enhance fruit acidity (Liu et al., 2012, 2013, 2018; Dima et al., 2014). Reportedly, carotenoid accumulation are associated with flesh color development (Tadmor et al., 2005; Bang et al., 2007, 2010). The aforementioned phytochemical compounds condition the overall nutritional compositions, eating quality, and fruit ripening (Liu et al., 2012; Dima et al., 2014; Ramirez et al., 2020; Wang C. et al., 2021). Fruit hormones such as ethylene and abscisic acid, and flesh composition (i.e., cellulose, hemicellulose, and pectin) determine fruit ripening, shelf-life, and chemical compositions influencing product quality such as fruit firmness, flesh- chewiness and -gumminess, and maturity (Gao et al., 2020; Sun et al., 2020; Anees et al., 2021).

Watermelon’s fruit quality are affected by polygenes, the growing environment and their interaction. Furthermore, fruit quality is a highly complex trait governed by physiological, biochemical and molecular processes (Yu et al., 2012; Guo et al., 2015; Fang et al., 2020; Sun et al., 2020). The genetic mechanism of fruit quality in watermelon was revealed through comparative transcriptome analysis (Grassi et al., 2013; Guo et al., 2015; Fang et al., 2020). The reports indicated that multiple genes are involved and differentially expressed, influencing fruit quality (Wechter et al., 2008; Kang et al., 2010; Guo et al., 2015; Wang et al., 2017; Zhu et al., 2017; Yue et al., 2021). Quantitative trait loci (QTL) analysis revealed that fruit quality influencing genes in watermelon are located across the 11 chromosomes (Sandlin et al., 2012; Ren et al., 2014; Cheng et al., 2016; Branham et al., 2017; Fall et al., 2019; Yang et al., 2021). The QTL were successfully used in marker-assisted breeding, including the parental selection and development of novel progenies of watermelon (Cheng et al., 2016; Shang et al., 2016; Branham et al., 2017; Zhang et al., 2018; Jiang et al., 2019; Jin et al., 2019; Yang et al., 2021).

Fruit traits of sweet watermelon are crucial for new product development and commercialization. The novel and value-added genotypes will serve the fresh market, processing and pharmaceutical industry. Value-added products (i.e., pickles, jam, fruit puree, popsicles, and watermelon juice) are derived from newly designed cultivars with good quality attributes. The new cultivars should possess balanced bioactive compounds of nutritive and therapeutic significance (Tlili et al., 2011a,b; Rico et al., 2020; Zamuz et al., 2021; Zia et al., 2021).

Watermelon breeders have relied on conventional selection methods (e.g., single plant, pure line, mass selection) to develop cultivars with excellent fruit quality traits (Davis and King, 2007; Davis et al., 2008; Gao et al., 2018a). However, the low genetic base of sweet watermelon (Levi et al., 2001a,^2004^; Che et al., 2003; Kwon et al., 2010; Solmaz et al., 2010; Hwang et al., 2011; Minsart et al., 2011; Sheng et al., 2012; Yang et al., 2016; Zhang et al., 2016; Singh et al., 2017; Stone et al., 2019) poses challenges for quality breeding. Also, in many watermelon improvement programs, a limited number of watermelon genotypes are utilized as breeding parents (Levi et al., 2001a,^2004^; Zhang et al., 2012; Pal et al., 2020). Most parental genotypes exhibit excellent agronomic and horticultural traits but share similar genetic backgrounds presenting genetic bottlenecks for breeding (Levi et al., 2001a; Guo et al., 2012; Wang et al., 2015; Zhang et al., 2016). Non-conventional breeding using gene-editing technologies presents a viable and alternative approach for speed breeding and release of watermelon varieties possessing suitable agronomic and quality traits to serve the value chains of the crop. For example, targeted editing of genes associated with agronomic traits in the watermelon using the clustered regularly interspaced short palindromic repeats (CRISPR/Cas9) enabled the selection of novel cultivars (Tian et al., 2017; Zhang et al., 2020a; Wang Y. et al., 2021). Furthermore, the gene editing and associated technologies will deliver knowledge on the genetic regulation of fruit quality traits in watermelon that will aid in the development of improved cultivars with enhanced nutritional compositions, market-preferred traits and extended storage life.

In light of the above background, this review aims to present the opportunities and progress made on the genetic analysis of fruit quality traits in watermelon as a guide for quality breeding based on economic and end-user attributes. The first section of the review highlights the genetic mechanisms involved in the biosynthesis of phytochemical compounds, phytohormones and fruit flesh structural components during watermelon fruit development and ripening. This is followed by a summary of the progresses made on molecular markers and QTL analysis for fruit quality attributes. The review presents the new generation genetic and breeding technologies and innovations for accelerated cultivar development with excellent fruit quality traits. The paper presented possible gene actions conditioning fruit ripening in citron watermelon genetic resources to guide current and future breeding. Information presented in this review is useful for watermelon variety design, product profiling and development to serve diverse value chains of the crop.

## Genetic Analysis of Fruit Quality in Sweet Watermelon

### Sugar Composition of Sweet Watermelon Fruit

Flesh sweetness is an important attribute that determines the eating quality of ripe watermelon fruit (Yativ et al., 2010; Liu et al., 2013). Sweetness is determined by the composition of the following sugars, namely: sucrose, fructose, and glucose (Ren et al., 2014; Zhu et al., 2017). The most important enzymes that regulate sugar metabolism of watermelon are sucrose synthase and sucrose phosphate synthase (Yativ et al., 2010; Guo et al., 2015). Also, the expression of sugar biosynthetic gene clusters including α-galactosidase, invertase and Urease diphosphate (UDP)-galactose/glucose pyrophosphorylase (UDP-Gal/Glc PPase) increase during watermelon fruit ripening. Reportedly, the gene designated as *Cla013902* influence sugar metabolism in watermelon (Guo et al., 2015). A total of nine α-galactosidase genes were identified in the watermelon genome. The nine genes are reportedly involved in the hydrolyzes of stachyose and raffinose (Guo et al., 2012).

Raffinose family oligosaccharides (RFOs), including raffinose and stachyose are the main photo-assimilates translocated in the phloem of cucurbit crops (Zhang et al., 2010). The hydrolysis of RFOs is crucial for sugar accumulation and transportation in the fruit. RFOs are hydrolyzed rapidly by α-galactosidase genes in the phloem. Sugar accumulation in the phloem and sucrose translocation to fruits require the action of insoluble acid invertase (Guo et al., 2012). Five insoluble acid invertase (IAI) genes are involved in sugar accumulation in watermelon (Guo et al., 2015). Of these, the IAI gene *Cla020872* is involved in the extracellular sucrose degeneration and thus facilitate fructose and glucose translocation and the intercellular sugar accumulation in watermelon fruit (Liu et al., 2013; Guo et al., 2015). Another gene *Cla013902*, also contribute to sugar metabolism in watermelon. Two major sugar transporter genes, namely: *Cla015835* and *Cla015836* are associated with sugar transportation and accumulation in watermelon fruit (Liu et al., 2013). Recently, the alkaline α-galactosidase gene *ClAGA2* expressed in the vascular bundle was identified as a major gene controlling stachyose and raffinose hydrolysis in watermelon. *ClAGA2* controls fruit raffinose hydrolysis and reduces sugar content in ripened watermelon fruits (Ren et al., 2021). Sugar Transporter 3 gene designed as *ClSWEET3* and Tonoplast Sugar Transporter gene (i.e., *ClTST2*) influence sugar transport and storage in watermelon fruit cell vacuoles (Ren et al., 2021). Sugar transporters genes denominated as *EDR6 (Cla97C05G087120)* and *STP (Cla97C01G018840)* were highly expressed during watermelon fruit ripening simultaneously influencing sugar accumulation and transportation (Umer et al., 2020b). A tonoplast sugar transporter gene designated as *ClTST2* (gene ID: *Cla000264*) was highly expressed during fruit ripening associating with tonoplast uptake and accumulation of sugars in watermelon fruit flesh cells (Ren et al., 2018). The gene *WMFBPA-2* was found to be influencing fructose breakdown (Umer et al., 2020a). Fructokinase (FK), fructose bisphosphate aldolase (FBA), and fructose 1-6, bisphosphate (FBP) genes regulate fructose metabolism of watermelon. The FK gene designated as *Cla97C05G094630*, *FBA* (*Cla97C04G076620*) and *FBP* (*Cla97C08G147060*) were the most important regulators of fructose metabolism in watermelon fruit (Umer et al., 2020b). The following genes: *Cla97C01G000640* (*SWEET*), *Cla97C05G087120* (*EDR6*), and *Cla97C01G018840* identified from sweet and sweet-and-sour genotypes reportedly regulate glucose biosynthesis, while *Cla97C03G064990* condition sucrose biosynthesis in watermelon (Umer et al., 2020b). A vacuolar sugar transporter gene named *ClVST1* was highly expressed during the ripening of watermelon fruit associated with sucrose accumulation (Ren et al., 2020). Genes involved in sugar biosynthesis can be up- or down-regulated during the breeding of desired genotypes. The sweetness product profile influences the needs and preferences of consumers and the industry during the development of value-added products.

### Amino Acids Profiles of Sweet Watermelon Fruit

Citrulline is the most abundant amino acid present in ripened watermelon fruit (Joshi et al., 2019; Zhong et al., 2019). Citrulline is a non-essential amino acid and is biosynthesized as a metabolic intermediate compound during the urea cycle (Bahri et al., 2013). This amino acid is a precursor of arginine, which is another major amino acid in watermelon fruit (Joshi et al., 2019; Song et al., 2020). Several genes involved in citrulline metabolism have been identified in watermelon. Of these, ornithine carbamoyltransferase (*OTC*; *ClCG05G018820*), *N*-acetylornithine aminotransferase (*N-AOA*; *ClCG09G003180*), *N*-acetylornithine-glutamate acetyltransferase (*N-AOGA*), *-1* (*ClCG11G013120*) and *CPS-2* (*ClCG09G021680*), *N*-acetylornithine deacetylase (*AOD-1), N*-acetylornithine deacetylase (*AOD-3)*, and Nitric-oxide Synthase gene (*ClCG01G004960*) are associated with citrulline biosynthesis in watermelon (Joshi et al., 2019). In addition, genes namely: *ASS-1*,*2*,*3*-Argininosuccinate synthase; *ASL-1*,*2-*Argininosuccinate lyase; *ARG* – Arginase; *ODC –* Ornithine decarboxylase; and *ADC-*Arginine decarboxylase are reportedly associated with citrulline catabolism (Joshi et al., 2019). The genes *Cla022154* (Argininosuccinate lyase), *Cla022273* (Nacetylglutamate Kinase), and *Cla022392* (Ornithine decarboxylase) are involved in citrulline biosynthesis, whereas candidate genes *Cla020511, Cla020512*, and *Cla020781* (Ornithine carbamoyltransferase) are involved in the biosynthesis and accumulation of arginine (Fall et al., 2019).

### Organic Acids Composition of Sweet Watermelon Fruit

The composition of organic acids enhances flesh acidity and improve the taste of ripe watermelon fruit. Organic acids including malic, quinic, citric, oxalic, and tartaric acids, accumulate in watermelon fruit (Liu et al., 2012; Gao et al., 2018a,b). Of these, citric and malic acids are abundantly present (Gao et al., 2018a,b). Differentially expressed genes, including the NAD-dependent malate dehydrogenase (NAD-cyt MDH), aluminum-activated malate transporter (ALMT), and citrate synthase (CS) influence the accumulation of organic acids in watermelon (Gao et al., 2018a). NAD-dependent malate dehydrogenase (NAD-cyt MDH) gene catalyzes the reversible conversion of malate into oxaloacetate (OAA) (Yao et al., 2011), while citrate synthase (CS) regulates the biosynthesis of citric acid. Aluminum activated malate transporters, and malate dehydrogenase genes regulate malate regulation and degradation (Umer et al., 2020a). The citrate synthase gene (*Cla97C03G068240*) and isocitrate dehydrogenase gene (*Cla97C01G008870*) are reportedly involved in citric acid synthesis and degradation (Umer et al., 2020b). Higher expression of malate dehydrogenases genes (i.e., *Cla008235* and *Cla011268*) and citrate synthases gene (*Cla013500*) are associated with enhanced accumulation of malic and citric acids (Gao et al., 2018a). Also, three other genes, namely: *WMALMT-3, WMMDH-1*, and *WMMDH-2*, which are highly expressed during watermelon fruit development and ripening reportedly regulate malate biosynthesis and catabolism (Umer et al., 2020a). *WMALMT-3* is the major gene regulating malic acid biosynthesis (Umer et al., 2020a). The gene denoted as *WMCS* is believed to affect the production and accumulation of citric acid (Umer et al., 2020a). The malate synthase gene *Cla97C03G054690* conditions malate synthesis and found to be more highly expressed in a sweet-and-sour watermelon genotype than in sweet watermelon genotype (Umer et al., 2020b). Also, the malate dehydrogenase genes, namely*: Cla97C06G125760* and *Cla97C05G103110*, and transmembrane malate transport gene (*Cla97C02G049340*), probably influence malate biosynthesis in watermelon (Umer et al., 2020b). Pyruvate kinase genes *Cla97C04G076530* and *Cla97C11G218660* are reportedly associated with malate breakdown during watermelon fruit ripening (Umer et al., 2020b). Gao et al. (2018a) bred sweet-and-sour watermelon cultivar designed as “SW” through a conventional breeding approach involving the crossing of sweet and citron watermelon genotypes. This finding suggests the possibility of improving organic acid content through targeted mutagenesis using gene-editing technologies such as Targeting Induced Local Lesions in Genomes (TILLING) and through gene editing using the clustered regularly interspaced short palindromic repeats (CRISPR)/CRISPR-associated system 9 (CRISPR/Cas9).

### Carotenoids Contents of Sweet Watermelon Fruit

The development of watermelon fruit flesh color is determined by the accumulation of various carotenoids. The fruit flesh exhibits a wide range of colors, including canary yellow, salmon-yellow, orange, red, coral-red, and white (Henderson et al., 1998; Guner and Wehner, 2003, 2004). Lycopene is the major carotenoid present in red- and pink-fleshed watermelon types (Tadmor et al., 2005; Kang et al., 2010). Yellow-fleshed watermelon contains mainly violaxanthin and neoxanthin, whereas orange-fleshed watermelon contains mainly β-carotene (Tadmor et al., 2005; Lewinsohn et al., 2006; Bang et al., 2007, 2010; Liu et al., 2012). White-fleshed watermelons accumulate the colorless phytofluene and light-yellow ζ-carotene (Kang et al., 2010; Grassi et al., 2013). The genetic variation in watermelon fruit flesh color suggests the presence of different mechanisms regulating carotenoid metabolism. Hence, understanding the mechanism of carotenoid inheritance allows for cultivar design with enhanced compositions of phytochemical compounds. The genome-wide comparative expression analysis of the carotenoid biosynthesis and metabolism pathway genes revealed complex gene expression and regulatory networks resulting in various carotenoids accumulation in watermelon fruit (Kang et al., 2010; Guo et al., 2015; Lv et al., 2015; Dou et al., 2017; Fang et al., 2020). Guo et al. (2015) identified a total of 26 genes responsible for carotenoid metabolism in watermelon, of which the phytoene synthase, β-carotene hydroxylase, 9-*cis*-epoxycarotenoid dioxygenase and carotenoid cleavage dioxygenase genes were the key biosynthesis genes. The phytoene synthase gene *Cla009122* is associated with white-flesh color development (Grassi et al., 2013; Guo et al., 2015). A chromoplast phosphate transporter gene *ClPHT4;2* (gene ID: *Cla017962*) plays a dominant role in flesh color formation in watermelon (Zhang et al., 2017). β-carotene hydroxylase genes, namely: *Cla006149* and *Cla011420* regulate β-carotene concentration. 9-*cis*-epoxy-carotenoid dioxygenase (NCED) is involved in the catabolic process, which converts 9-*cis*-violaxanthin or 9-*cis*-neoxanthin to xanthoxin (Schwartz et al., 2003). NCED genes, namely: *Cla005404* and *Cla009779*, facilitate carotenoid catabolism to initiate the biosynthesis of abscisic acid [ABA] (Li et al., 2012; Guo et al., 2015). Carotenoid cleavage dioxygenase (CCD) catalyses the oxidative cleavage of carotenoids and leads to the production of apocarotenoids (Guo et al., 2015). Apocarotenoids, including β-cyclocitral, β-ionone, geranial, geranyl acetone, theaspirone, α-damascenone and β-damascenone contribute to fruit flavor and aroma. The CCD gene *Cla015245* serves as an important link between flesh pigmentation and flavor (Guo et al., 2015). A recent study indicated that the gene *Cla018406* is responsible for β-carotene accumulation in orange flesh-colored fruit in watermelon (Wang et al., 2021). Also, the gene *Cla007686* was reportedly highly expressed in the red flesh-colored watermelon, serving as a key regulator of lycopene accumulation. The genes designated as *Cla018767*, *Cla018768*, *Cla018769, Cla018770, and Cla018771* were linked to controlling red-flesh color development in watermelon for being involved in lycopene biosynthesis (Li et al., 2020). The genes *Cla003760* and *Cla021635* conferred yellow flesh color development (Yuan et al., 2021). Carotenoid metabolic pathway genes namely: *ClPSY1* (*Cla009122*), *ClZDS* (*Cla003751*), *ClNCED-7* (*Cla005404*), *ClCRTISO* (*Cla017593*), *ClCHYB* (*Cla006149*), *ClLCYB* (*Cla005011*), *ClPDS* (*Cla010898*), *ClNCED-1* (*Cla009779*), and *ClCHXE* (*Cla011420*) are involved in the development of white and pale-yellow fruit flesh color of watermelon (Wang et al., 2021). Of these genes, *ClPSY*, *ClLCYB*, *ClCHYB*, *ClZEP*, and *ClNCED* regulate the biosynthesis of lycopene, carotene, lutein, zeaxanthin and neoxanthin, respectively (Kang et al., 2010; Lv et al., 2015; Dou et al., 2017; Wang C. et al., 2021). *ClPsy1* (gene ID *Cla97C01G008760*) was recently identified as the major gene conditioning the golden flesh color in watermelon (Liu et al., 2022). Previous studies also identified the lycopene β-cyclase gene *ClLCYB* (i.e., *Cla005011*) for being the main gene conditioning the development of red-flesh color in watermelon (Wang et al., 2016, 2019; Li et al., 2020), and in the development of canary yellow flesh-color (Bang et al., 2007, 2014). The down-regulation of *ClLCYB* (gene ID: *Cla97C04G070940*) is associated with changes from pale-yellow to red flesh, whereas overexpression of *ClLCYB* causes color change from red to orange (Zhang et al., 2020b). Also, phytoene synthase gene *ClPSY1* promotes phytofluene accumulation, thus influencing the total carotenoid content composition of ripened watermelon fruit (Fang et al., 2020). High contents of phytoene were found to be highly correlated with high β-carotene contents in watermelon (Fang et al., 2020), suggesting that phytoene is a key compound for carotenoid metabolism in watermelon. Plastid lipid-associated genes (i.e., *ClPAPs*, *Cla006670*, *Cla010946, Cla008831, Cla014416, Cla021506, Cla003468*, and *Cla003198*) are reportedly involved in the formation of plastoglobules, globular and crystalloid chromoplasts (Fang et al., 2020). Watermelon breeders aiming to improve carotenoid profiles should select progenies with genes conditioning the carotenoid biosynthesis pathway.

### Volatile Compounds of Sweet Watermelon Fruit

Volatile compounds determine the aroma and flavor of the ripened watermelon fruit (Dima et al., 2014; Fredes et al., 2017; Liu et al., 2018; Bianchi et al., 2020). VOCs are synthesized via the methylerythritol phosphate or mevalonate pathways which result in the accumulation of terpenoids, and C6 and C9 alcohols/aldehydes (ADHs), ethyl acetate, acetaldehyde, tetradecanoic acid and methyl acetate (Pino et al., 2003; Dudareva et al., 2013; Wei et al., 2016). Various VOCs, including monoterpenes, apo-carotenoids, ketones, aldehydes, alcohols, acids, olefins, and furans, accumulate in the watermelon fruit (Lewinsohn et al., 2006; Liu et al., 2012; Bianchi et al., 2020). A recent study by Bianchi et al. (2020) indicated that C-9 aldehydes and alcohols were the prevalent VOCs present in ripened watermelon fruit. VOCs such as 4-oxononanal and 2- hydroxy-, 2- pentyloxy-, 2- hexyloxy-, 2- octyloxy-, 2- nonyloxy-, 2-[(*Z*)-3- noneyloxy]-, 2-[(*Z*)-2- nonenyloxy]-, 2-[(*Z,Z*)-3,6- nonadienyloxy]-, 2-benzyl- oxy-, and 2-phenetyloxy-5-pentyltetrahydrofurans were identified as key flavor influencing compounds in watermelon fruit (Yajima et al., 1985). Also, β-Ionone, dihydroactinodiolide, and β-cyclocitral were found to be prevalent in ripened watermelon fruit (Lewinsohn et al., 2006). VOCs such as 2-pentyl-furan, (*E*)-2-(2-pentenyl) furan, 2-ethyl-1-hexanol; nonanal, (*E*)-2-nonenal, nonanol, 2-nonen-1-ol; Nol-3, (Z)-3-nonen-1-ol, 2,6-nonadien-1-ol, (*E,Z*)-3,6-nonadien-1-ol, nonanoic acid, 1,10-undecadiene; (*E*)-geranyl acetone; limonene, and β-ionone were reported to be accumulated in watermelon fruit (Liu et al., 2012). VOCs such hexanal, 6-methyl-5-hepten-2-one, (*E*)-2-octenal, 4-nonenal, (*Z*)-6-nonenal, Nonanal, (*Z*)-3-Nonen-1-ol and (*E,Z*)-2,6-nonadienal, (*Z,Z*)-3,6-nonadien-1-ol, (*E*)-2-nonenal and 1-nonanol were identified in seedless watermelon fruit, with 3-nonen-1-ol/(*E,Z*)-2,6-nonadienal (16.5-28.2%), (*E*)-2-nonenal (10.6–22.5%), (*Z*)-6-nonenal (2.0-11.3%) and hexanal (37.7%) being the abundant flavor and aroma enhancing compounds (Beaulieu and Lea, 2006). (*Z*)-3-nonen-1-ol, (*E*)-2-nonenal and (*Z*,*Z*)-3,6-nonadien-1-ol were also the predominant VOCs in mini-watermelon fruits (Dima et al., 2014). Additionally, (*Z*)-3-Nonen-1-ol, 2-ethyl-1-hexanol, nonanal, (*E,Z*)-3,6-nonadien-1-ol, and nonanol were reportedly dominant VOCs accounting for 81–87% in red-fleshed watermelon and 75–79% in yellow and orange-fleshed watermelon (Liu et al., 2012). Off-flavor VOCs were reported in watermelon, including (*E*)-2-heptenal, decanal, octanol, diisopropyl disulfide, hexanol, (*E*)-2-decenal, and (*E*)-2-octenol (Yang et al., 2020).

Sensory evaluation revealed that flavor is a vital fruit quality attribute relating to the refreshing perception of watermelon, an attribute linked with VOCs (Ramirez et al., 2020; Mandha et al., 2021). Comparative transcriptome analysis revealed ADHs genes: *Cla97C01G013600, Cla97C05G089700, Cla97C01G001290 Cla97C05G095170*, and *Cla97C06G118330* enhanced flavor and aroma formation of ripened watermelon fruit (Gong et al., 2021). The reported genes condition the biosynthesis of VOCs 2,6-nonadienal, (*E,Z*); 2-non-enal, (*E*)-; 3,6-nonadien-1-ol, (*E,Z*)-; and 3-nonen-1-ol, (*Z*)-, respectively (Gong et al., 2021). Previous findings pinpointed that VOCs are main flavor-forming compounds in watermelon (Liu et al., 2012; Dima et al., 2014). Other ADHs genes such as *Cla97C09G172570*, *Cla97C05G092340*, *Cla97C01G001290*, and *Cla97C08G152400* and lipoxygenase (LOX) gene (*Cla97C06G115570*) are reportedly linked to aroma and flavor development of the ripened watermelon fruit (Gong et al., 2021). Understanding the genes involved and its expressions in aroma and flavor development of watermelon fruit is useful in variety development.

### Phytohormones of Sweet Watermelon Fruit

Phytohormones such as ethylene and ABA regulate watermelon fruit ripening and tissue degradation, thus influencing fruit quality and shelf life (Zhou et al., 2016; Wang et al., 2017). Transcriptome analysis revealed 22 genes conditioning ethylene biosynthesis and signaling pathway including ACC synthase (ACS), ACC oxidase (ACO), ethylene receptor (ETR), and ethylene-responsive factor (ERF). The genes regulate the production and accumulation of phytohormones in watermelon (Guo et al., 2015). ACS and ACO are key genes regulating ethylene biosynthesis (Guo et al., 2015). The ACS gene denoted as *Cla011522* is highly expressed during watermelon fruit development and ripening, making it the most important gene regulating ethylene biosynthesis. The following ACS genes: *Cla000483, Cla006245, Cla006634, Cla011230, Cla014057, Cla014652*, and *Cla022653* showed a relatively low expression levels playing vital roles in ethylene biosynthesis (Guo et al., 2015). ACO genes namely: *Cla018620* and *Cla018621* are reportedly involved in regulating endogenous ethylene biosynthesis (Guo et al., 2015). *ACS* (i.e., *Cla022653* and *Cla011522*), and *ACO* (i.e., *Cla014827* and *Cla016287*) genes induce increased ethylene accumulation in watermelon fruit (Zhou et al., 2016). An ethylene receptor gene *Cla021550* with low expression during fruit development and ripening is associated with decreased ethylene biosynthesis (Guo et al., 2015).

Watermelon flesh firmness is influenced by the levels of phytohormones, especially ABA (Wang et al., 2017). ABA concentrations in the flesh of sweet dessert watermelon genotype “97103” and egusi watermelon (*C. mucosospermus*) line “PI179878” increased during fruit development and ripening, but low concentrations were observed in citron watermelon accession “PI 296341-FR” (Wang et al., 2017). Reduced ABA concentrations is believed to be associated with hard-flesh, reduced tissue breakdown and extended shelf and storage life in watermelon. The following genes are involved in ABA biosynthesis in watermelon: *Cla009779* (*NCED*), *Cla005404* (*NCED*), *Cla020673* (*CYP707A*), *Cla006655* (*UGT*), and *Cla020180* (*SnRK2*) (Wang et al., 2017). Of these, *Cla009779*, *Cla005404*, and *Cla005457* enhanced ABA accumulation. A recent report indicated that the genes *Cla016033* and *Cla012507* controlled ABA biosynthesis in watermelon (Sun et al., 2020). The high correlation between ABA and total sugar content suggests the involvement of ABA in regulating watermelon fruit quality via sugar metabolism (Wang et al., 2017).

### Fruit Flesh Texture of Sweet Watermelon Fruit

Fruit flesh texture properties, especially flesh firmness, influence sensory quality and affect taste, flavor, and the shelf life of ripened watermelon fruit (Gao et al., 2020; Sun et al., 2020). A hard flesh results in reduced juice and poor flavor, whereas a soft flesh shows poor eating quality, storage and reduced shelf life (Gao et al., 2020). Changes in flesh firmness are mainly attributed to changes in fruit flesh structural components. Cellulose, pectin and hemicellulose are the main components of the cell wall, which largely determine the firmness and sensory qualities of watermelon fruit flesh (Guo et al., 2015; Gao et al., 2020; Sun et al., 2020; Anees et al., 2021). The catabolism of fruit flesh structural components (i.e., pectin, cellulose, and hemicellulose) cause watermelon fruit softening (Gao et al., 2020). Comparative transcriptome analysis revealed the gene *Cla016033* influencing the components of the cell wall and flesh firmness. Also, the gene designated as *Cla012507* is responsible for hard flesh texture of watermelon fruit (Gao et al., 2020). Transcriptome analysis based on citron watermelon accession “PI186490” and sweet watermelon ‘Sanbai’ revealed 85 genes including 10 pectinesterase genes, 2 polygalacturonase genes, 4 pectate lyase genes, 10 glycosyltransferase genes, 9 cellulose synthase genes, 8 β-galactosidase genes, 9 xyloglucan transferase genes, and 2 lipoxygenase genes were up-regulated. The listed genes influence flesh texture by strengthening the cell wall structure of watermelon fruit (Gao et al., 2020). *Cla11211, Cla10077, Cla14927, Cla18814, Cla12351*, and *Cla002537* were highly expressed during ripening, enhancing watermelon flesh textural properties (Gao et al., 2020). Peroxidases (i.e., *Cla003188*, *Cla003189*, *Cla014013*, *Cla019382*, and *Cla003187*), transcription factors genes (i.e., *Cla009283*, *Cla012465*, *Cla015515*, *Cla020969*, *Cla022083*, *Cla013612*, *Cla016224*, *Cla009263*, *Cla013099*, *Cla007307*, *Cla007656*, and *Cla018059*) and phytohormone biosynthesis genes (i.e., *Cla006604*, *Cla004102*, *Cla014808*, *Cla019806*, *Cla020976*, and *Cla021550*) strengthened the cell wall structure of the ripe watermelon fruit (Gao et al., 2020). Gene expression analysis between watermelon hard flesh firmness lines (HWF) and soft flesh firmness lines provided three main gene networks, namely water-soluble pectin, cellulose, hemicellulose, and protopectin, responsible in the structural integrity of watermelon fruit (Anees et al., 2021). The genes *Cla012351* (Cellulose synthase) and *Cla004251* (Pectinesterase) influence cellulose and protopectin contents, whereas *Cla004120*, *Cla009966* (Cellulose synthase), and *Cla006648* (Galactosyltransferase) enhanced the accumulation of water-soluble pectin, cellulose, and protopectin in the watermelon flesh. Also, the following genes: *Cla007092*, *Cla004119*, and *Cla018816*, influence the composition of hemicellulose, cellulose, and protopectin, respectively (Anees et al., 2021). Ethylene accumulation influence fruit softening in watermelon, making the fruit susceptible to cracking and rotting. Ethylene-responsive transcription factor 4 gene *ClERF4* conditioned hard watermelon rind (Liao et al., 2020). The textural quality of watermelon fruit is also influenced by rind hardness/firmness (Yang et al., 2021). Genes conditioning rind hardness are not co-localized with genes involved in fruit cracking (Sun et al., 2020). The following genes: *MDP*, *DHDDS*, *POD1*, and *GST* are involved in reduced fruit cracking, whereas *CHDH*, *GPAT*, *POD2*, *SMT*, *KCS*, *XET1*, *XET2*, and *DHCR24* promote watermelon fruit cracking (Jiang et al., 2019). Genes influencing rind hardness and fruit cracking should be integrated in watermelon breeding to improve quality and shelf life of ripened watermelon fruit.

## Molecular Markers and Quantitative Trait Loci Analysis of Phytochemical Compounds, Phytohormones and Fruit Flesh-Structural Compounds in Sweet Watermelon

### Molecular Markers Reported in Genetic Analysis of Watermelon

Molecular markers are complementary tools to phenotyping, allowing accelerated cultivar development and deployment. Molecular markers such as simple sequence repeat (SSR) or microsatellites, insertion-deletion (InDel), single nucleotide polymorphism (SNP), kompetitive allele-specific polymorphism (KASP) and cleaved amplified polymorphic sequence (CAPS) have been developed to genotype watermelon populations for fruit quality breeding (Liu et al., 2015; Jin et al., 2019; Subburaj et al., 2019; Li et al., 2020). For example, InDel markers InDel27_fc6 and InDel28_fc6 were developed for marker-assisted selection for scarlet red flesh color (Li et al., 2020). A SNP marker linked to the lycopene b-cyclase gene (*LCYB*) has been developed for genotype selection (Bang et al., 2007). Kompetitive allele-specific polymorphism markers co-segregating with pale flesh color phenotype have been developed for genetic analysis of watermelon (Pei et al., 2021). The KASP marker (*chr06_7040350*) effectively distinguished white and pale-yellow fleshed watermelons useful for marker-assisted breeding (Wang et al., 2017). A cleaved amplified polymorphic sequence marker (CAPS-C^1976^) was developed for distinguishing yellow and orange-fleshed watermelon genotypes (Jin et al., 2019). A CAPS marker was also developed to effectively select watermelon with canary yellow and red fruit colors (Bang et al., 2007). Simple sequence repeat marker (BVWS00954) was identified on chromosome 6 conditioning watermelon flesh firmness (Gao et al., 2016). There are limited molecular markers developed and reported for fruit quality traits in sweet watermelon genetic resources. It is essential to develop diagnostic molecular markers to aid efficient screening and selection of desirable parents for fruit quality improvement and complement phenotypic selection.

### Quantitative Trait Loci Analysis of Sweet Watermelon Fruit Quality

Next-generation sequencing (NGS) technologies have allowed genome sequencing and identification of QTL associated with watermelon fruit quality and other important agronomic and horticultural traits (Table 1). Sugar transporter genes *Cla015835* and *Cla015836* are located at the region of the major sugar content QTL *Qbrix2-2* on chromosome 2 in watermelon (Ren et al., 2014). Two QTL (*Qbrix2-1* and *Qsuc2-1*) linked to sucrose content were mapped on chromosome 2 (Ren et al., 2014). Fructose QTL *Qfru2-1* and *Qfru2-2* are co-localized with QTL associated with total sugar content, namely: *Qbrix2-1* and *Qbrix2-2* (Ren et al., 2014). The glucose QTL *Qglu6* was co-localized with the QTL conditioning fructose content (Ren et al., 2014). Two QTL controlling sucrose content, namely: *Qsur2-1* and *Qsur2-2* were mapped on chromosomal 2 linked with brix influencing QTL *Qbrix2-1* and *Qbrix2-2* and influencing fructose levels such as QTLs *Qfru2-1* and *Qfru2-2* (Ren et al., 2014). Two QTL, *Qsur5-2 and Qsur8-1* localized on chromosomes 5 and 8, respectively, influence sucrose content in watermelon (Fall et al., 2019). Cheng et al. (2016) identified 4 QTL linked to sugar content in watermelon (Table 1). The following genes: *Cla021522* (phosphate permease), *Cla021325* (pectinesterase), *Cla021640, Cla021693* (membrane transporters), *Cla021639, Cla021641, Cla021642, Cla021643* (sugar/inositol transporters), *Cla021807* (galactokinase), and *Cla021809* (neutral invertase) condition sugar biosynthesis. These genes were located on chromosome 5 (Guo et al., 2012; Zhu et al., 2017; Fall et al., 2019). A QTL affecting sugar content (*QBRX2-1*) correlated with a tonoplast sugar transporter 2 *ClTST2* and mapped on chromosome 2 (Ren et al., 2018). Major QTL affecting citrulline biosynthesis are localized on chromosomes 1, 8, and 11 of watermelon (Fall et al., 2019). The reported genes involved were *Cla022154* (Argininosuccinate lyase), *Cla022273* (Nacetylglutamate Kinase), and *Cla022392* (Ornithine decarboxylase). These genes were located on chromosome 8, while the additional gene *Cla006553* (Pyrroline-5-carboxylate synthase) was mapped on chromosome 11 (Fall et al., 2019). QTL conditioning arginine was located on chromosomes 2 and 5 of watermelon (Fall et al., 2019). The arginine candidate genes *Cla020511*, *Cla020512*, and *Cla020781* (Ornithine carbamoyltransferase) were localized on chromosome 5 (Fall et al., 2019). A QTL *LCYB4.1* related to lycopene content was mapped on chromosome 4 (Liu et al., 2015). Recently, the genomic region *Y^scr^* associated with the scarlet-red flesh color was mapped on chromosome 6 of watermelon (Li et al., 2020). A major QTL (*qFC*.*1*) linked to β-carotene in watermelon was identified on chromosome 1 (Branham et al., 2017). Also, the β-carotene hydroxylase *CHYB* gene (gene ID: *Cla011420*) was mapped on chromosome 1 (Jin et al., 2019). The QTL *qfc10.1* located on chromosome 10 provides the pale flesh color of watermelon (Pei et al., 2021). Carotenoid biosynthesis enzymes carotenoid isomerase gene (*CRTISO*; gene ID: *Cla017593*) is located on chromosome 10 (Jin et al., 2019). Primary genes controlling watermelon flesh hardness are located on chromosomes 2 and 8 (Sun et al., 2020). Chromosome 10 harbor QTL associated with rind hardness (Yang et al., 2021). QTL associated with rind hardness was mapped to chromosome 4 of watermelon (Hashizume et al., 2003).

**TABLE 1:** Reported quantitative trait loci (QTLs) associated with fruit quality traits in watermelon.

Fruit quality traits	QTL name	Population	PVE (%)	AdE	DomE	Chr	POC (cM)	References
Total soluble solutes	*brx7.1*	CLL × CLL	7.30	0.27	−	7	34.5	Sandlin et al., 2012
	*brx8.1*	CLL × CLL	7.00	–0.26	−	8	17.9	Sandlin et al., 2012
	*brx9.2*	CLL × CLL	12.10	0.40	−	9	76.7	Sandlin et al., 2012
	*brx9.1*	CLL × Egusi	21.60	0.65	0.03	9	0	Sandlin et al., 2012
	*Qbrx5-1*	CLL × CLL	12.40	0.37	−	5	189.6	Fall et al., 2019
	*Qbrx8-2*	CLL × CLL	13.40	–0.40	−	8	0	Fall et al., 2019
	*Qbrix2-1*	CLL × CLL, CLL × CLV, CLL × Egusi	22.14	0.83	−	2	15.9	Ren et al., 2014
	*Qbrix2-2*	CLL × CLL, CLL × CLV, CLL × Egusi	28.87	0.54	−	2	73.9	Ren et al., 2014
	*Qbrix6*	CLL × CLL, CLL × CLV, CLL × Egusi	8.28	0.41	−	6	35.5	Ren et al., 2014
	*BCC2.1 2*	CLL × CLL	2.72	0.40	0.27	2	24.31	Cheng et al., 2016
	*Qbrix8*	CLL × CLL, CLL × CLV, CLL × Egusi	13.71	0.79	−	8	24.3	Ren et al., 2014
	*BCC8.1*	CLL × CLL	6.87	–0.68	−	8	−	Liu et al., 2015
	*BCE2.1*	CLL × CLL	6.14	–0.52	−	1	−	Liu et al., 2015
	*BCE8.1*	CLL × CLL	5.27	–0.48	−	8	−	Liu et al., 2015
Fructose	*Qfru5-1*	CLL × CLL	18.30	–3.63	−	5	16.9	Fall et al., 2019
	*Qfru5-2*	CLL × CLL	18.30	–3.11	−	5	45.4	Fall et al., 2019
	*Qfru2-1*	CLL × CLL	25.95	3.30	−	2	17.9	Ren et al., 2014
	*Qfru2-2*	CLL × CLL, CLL × CLV, CLL × Egusi	24.55	2.11	−	2	75.9	Ren et al., 2014
	*Qfru3*	CLL × CLL, CLL × CLV, CLL × Egusi	5.86	1.12	−	3	64.7	Ren et al., 2014
	*Qfru6*	CLL × CLL, CLL × CLV, CLL × Egusi	15.93	2.33	−	6	23.8	Ren et al., 2014
	*Qfru8*	CLL × CLL, CLL × CLV, CLL × Egusi	14.90	3.52	−	8	25.3	Ren et al., 2014
	*FCE10.1 10*	CLL × CLL	11.79	–0.82	0.29	10	116.51	Cheng et al., 2016
Glucose	*Qglu6*	CLL × CLL, CLL × CLV, CLL × Egusi	16.71	2.51	−	6	23.8	Ren et al., 2014
	*Qglu5-1*	CLL × CLL	12.80	–2.67	−	5	17.9	Fall et al., 2019
	*Qglu5-2*	CLL × CLL	9.60	–2.16	−	5	45.4	Fall et al., 2019
	*Qglu5-3*	CLL × CLL	11.90	–2.60	−	5	66.6	Fall et al., 2019
	*Qglu8-1*	CLL × CLL	17.40	3.01	−	8	49.6	Fall et al., 2019
	*Qglu8-2*	CLL × CLL	14.50	–2.86	−	8	135.5	Fall et al., 2019
	*GCE1.1 1*	CLL × CLL	6.49	0.29	-2.39	1	4.01	Cheng et al., 2016
Sucrose	*Qsur5-2*	CLL × CLL	28.30	0.02		5	44.8	Fall et al., 2019
	*Qsur8-1*	CLL × CLL	8.80	–0.02		8	50.6	Fall et al., 2019
	*Qsur2-1*	CLL × CLL, CLL × CLV, CLL × Egusi	6.39	0.82		2	15.9	Ren et al., 2014
	*Qsur2-2*	CLL × CLL, CLL × CLV, CLL × Egusi	9.65	0.76		2	71.9	Ren et al., 2014
	*Qsur5*	CLL × CLL, CLL × CLV, CLL × Egusi	0.54	7.97		5	19.1	Ren et al., 2014
	*SCE1.1 1*	CLL × CLL	7.02	–0.03	1.19	1	19.01	Cheng et al., 2016
Lycopene	*Qlyc3-1*	CLL × CLL	18.50	0.68		3	31.8	Fall et al., 2019
	*Qlyc11-1*	CLL × CLL	9.70	–0.50		11	105.2	Fall et al., 2019
	*FC4.1*	CLL × CLL, CLL × CLV	−	−		4	54	Zhang et al., 2017
	*Yscr*	CLL × CLL	−	−		6	−	Li et al., 2020
	*LCYB4.1*	CLL × CLL	83.50	19.79		4	−	Liu et al., 2015
β-Carotene	*qFC.1*	CLL × CLL	−	−		1	20	Branham et al., 2017
Flesh color	*FC4.1*	CLL × CLL	34.68	–0.01		4	−	Liu et al., 2015
	*FC3.1*	CLL × CLL	2.56	0.00	-0.28	3	−	Liu et al., 2015
	*FC6.1*	CLL × CLL	3.87	0.17	0.23	6	−	Liu et al., 2015
	*FC11.1*	CLL × CLL	5.16	–1.06	0.27	11	−	Liu et al., 2015
	*FCR1*	CLL × CLL	27.59	–0.49	0.22	10	−	Pei et al., 2021
	*FCR2*	CLL × CLL	28.58	–0.50	0.24	10	−	Pei et al., 2021
	*CC*	CLL × CLL	17.27	–0.01	0.00	10	−	Pei et al., 2021
Citrulline	*Qcit1-1*	CLL × CLL	11.50	27.06		1	67.7	Fall et al., 2019
	*Qcit8-1*	CLL × CLL	21.40	36.01		8	48.6	Fall et al., 2019
	*Qcit11-1*	CLL × CLL	12.90	–28.16		11	118.8	Fall et al., 2019
Arginine	*Qarg2-1*	CLL × CLL	15.70	11.96		2	79.7	Fall et al., 2019
	*Qarg5-1*	CLL × CLL	30.20	13.06		5	189.6	Fall et al., 2019
Rind hardness	*RH9*	CLL × CLL	5.44	60.15		9	257	Yang et al., 2021
	*RH10*	CLL × CLL	49.11	–297.56		10	44	Yang et al., 2021
Rind toughness	*RTO10*	CLL × CLL	10.33	–0.32		10	49	Yang et al., 2021
Rind thickness	*RTH2*	CLL × CLL	14.74	–0.20		2	214	Yang et al., 2021

*CLL, Citrullus lanatus var. lanatus; CLV, Citrullus lanatus var. citroides; PVE, phenotypic variation explained; AdE, additive effects; DomE, dominance effect; Chr, chromosome; POC, position of chromosome; cM, centimorgan, –, not available.*

Quantitative trait loci conditioning organic acids, VOCs, flesh and cell-wall structural compounds are yet to be discovered and mapped in watermelon. NGS technology combined with sequencing-based gene mining strategies such as bulk segregant analysis (BSA), bulked segregant analysis sequencing (BSA-seq), and genome-wide association study (GWAS) have been successfully used in gene discovery associated with agronomic and horticultural traits in watermelon (Wei et al., 2017; Dong et al., 2018, 2021; Ren et al., 2018; Zhu et al., 2019; Gimode et al., 2020; Cho et al., 2021; Liu et al., 2021; Wang C. et al., 2021; Wang et al., 2022). These technologies are useful for mining QTL linked to organic acids, VOCs, flesh and cell-wall structural compounds in watermelon. QTL mapping is based on whole-genome re-sequencing of DNA bulk samples of F_2_ generation derived from watermelon genotypes with the contrasting expression of organic acids, VOCs and cell-wall structural compounds. Genome-wide and bulk segregant analysis analyses can then be applied to detect genomic regions harboring genes associated with these compounds in watermelon. These will also facilitate the development of molecular markers for marker-assisted selection for developing watermelon genotypes with excellent fruit quality attributes. Overall, QTL linked to fruit quality traits provides opportunities for marker-assisted selection to breed watermelon genotypes with desired end-user quality attributes required by growers, consumers, and the industry.

## Gene Editing for Fruit Quality Traits in Sweet Watermelon

The genetic regulation of watermelon fruit quality has been unraveled through comparative transcriptome analysis involving comparative cultivars (Guo et al., 2012; Grassi et al., 2013; Gao et al., 2020; Sun et al., 2020). Previous studies revealed a complex gene network influencing watermelon fruit quality. Conventional breeding approaches have been widely used in watermelon breeding programs to develop cultivars with desirable fruit quality traits. For example, a watermelon cultivar “SW” with high total soluble sugar and organic acid contents was developed by crossing the sweet watermelon inbred line “203Z” and citron watermelon accession “PI 271769-FR” (Gao et al., 2018a). Sweet watermelon breeding lines “USVL-370” and “USVL-380” with low total soluble solutes (TSS) were developed from an F_2_ population through crosses of the citron watermelon accession PI 595203 and *C. lanatus* var. *lanatus* variety “New Hampshire Midget” (Levi et al., 2016; Levi and Ling, 2017). USVL-220 with TSS ranging from 8.5 to 11.0% was selected from an F_2_ population derived from a cross involving F_1_ hybrid sweet watermelon cultivar “New Hampshire Midget” × “Griffin 14113” (*C. lanatus* var. *citroides*) with *C. colocynthis* accession “PI386015” (Levi et al., 2011). In some instances, the development of sweet watermelon cultivars with desirable fruit quality attributes were derived from uncontrolled pollination. For example, a sweet watermelon cultivar “MSW-28” exhibiting medium sugar content, high lycopene content, and crisp flesh texture was selected from an F_6_ population derived from half-sib families of different varieties of commercial sweet watermelon and citron watermelon lines (Davis and King, 2007). Also, watermelon cultivars “LSW-177” and “LSW-194” with low fruit TSS content and firm texture and intense yellow flesh color were selected from the crosses of *C. lanatus* var. *lanatus* and *C. lanatus* var. *citroides* accessions (Davis et al., 2008).

Sweet dessert watermelon has a narrow genetic base attributed to many years of domestication and selection by growers for fruit quality traits such as red-scarlet flesh color and sweetness (Levi et al., 2001b; Hwang et al., 2011; Wu et al., 2019). These, in turn have resulted in limited genetic improvement of fruit quality attributes in modern cultivars. Genome editing has the potential to develop watermelon genotypes with desired fruit quality attributes (Tian et al., 2017). The clustered regularly interspaced short palindromic repeats (CRISPR)/CRISPR-associated system 9 (CRISPR/Cas9) is the widely used genome editing platform allowing for substantial improvement of economic traits. CRISPR/Cas9 was used for targeted mutagenesis of the phytoene desaturase gene *CIPDS*, which confers the albino phenotype in watermelon. CRISPR/Cas9 was used to induce mutations on the *ClPDS* gene sequences ATGCCGCTAGAGTGGTGCC and CGAGATGCTGTTCTCCATT. Transgenic watermelon plants developed using the technology harbored the *ClPDS* mutations and expressed the albino phenotype (Tian et al., 2017). CRISPR/Cas9 system was successfully employed to knockout the *CLPSK1* gene, encoding the PSK precursor, to confer enhanced resistance to *Fusarium oxysporum* f.sp. *niveum* in watermelon (Zhang et al., 2020b). Gynoecious (i.e., female flowers only) watermelon lines were derived using CRISPR/Cas9 system by targeted mutations of the gene sequences CTACTACTGGACTTAATAAGG and CCTTTGGACATGGCCATGCAGC (i.e., ATG and TGA arms, respectively) associated with the *ClWIP1* (gene ID *Cla008537*) (Zhang et al., 2020b). Also, CRISPR/Cas9-mediated mutagenesis of the *ClBG1* gene sequences (i.e., GAGGAAGATTCGCCACCGTTGG and CCGAGCGGAATTTGTTTTCGGAA) influencing size resulted in decreased watermelon seed size of transgenic plants (Wang Y. et al., 2021). Targeted mutagenesis of the watermelon acetolactate synthase (*ALS*) gene designated as *ClALS* (gene ID *Cla019277*) involved in the biosynthesis of the amino acidsvaline, leucine and isoleucine aided in the development of herbicide-resistance lines (Tian et al., 2018). This was achieved by CRISPR/Cas9-editing of the sequence GAAGTTCCGAGAAGAATGAT harboring the *ClALS* gene. Recently, Ren et al. (2021) reported that knocking out of sugar transporter genes *ClAGA2*, C*lSWEET3*, and *ClTST2* affected fruit sugar accumulation in watermelon. These reports indicate that CRISPR/Cas9 may offer opportunities for effective genome editing to develop watermelon genotypes with desired fruit quality attributes. Watermelon genotypes with desired fruit quality attributes can be developed through QTL analysis influencing the contents of sugars, lycopene, β-carotene, citrulline and arginine biosynthesis. These QTLs could be targeted for CRISPR/Cas9-mediated gene-editing to create a genetic variation for the selection and advancement of new progenies. Recently, editing of sucrose transporter gene *ClVST1* using CRISPR/Cas9 gene editing platform resulted in the development of watermelon lines with decreased sugar content, whereas overexpression of *ClVST1* increased sucrose content in the mutant lines (Ren et al., 2020). The development of an efficient CRISPR/Cas9 protocol is required for effective mutagenesis to improve watermelon fruit quality traits to facilitate product development for commercialization.

## Utilization of Citron Watermelon for Gene Discovery Associated With Fruit Quality

Citron watermelon belongs to the family Cucurbitaceae, a closely related species of sweet watermelon (Chomicki and Renner, 2015; Renner et al., 2017). Both species display similar phenotypic traits, including growth habit, flowering habit (i.e., monoecious) and fruit traits (i.e., fruit shape and rind stripe patterns). Flesh colors are similar in both species (i.e., white, yellow, and orange) except the deep red flesh color absent in citron watermelon (Levi et al., 2012; Ngwepe et al., 2019, 2021). Regarding flesh texture, sweet watermelon has a relatively soft flesh (1.0–1.8 kg/cm^2^) compared to citron watermelon, which has a medium-hard and crispy flesh (∼6.00 kg/cm^2^) texture (Fredes et al., 2017; García-Mendívil et al., 2019; Anees et al., 2021). This is attributed to the low expression levels of flesh texture related genes during fruit development (Guo et al., 2015). The mechanisms of fruit ripening at physiological and molecular levels differ between the two species (Guo et al., 2011, 2015; Zhou et al., 2016; Wang et al., 2017). Comparative transcriptome analysis between *C. lanatus var. lanatus* and *C. lanatus var. citroides* provided insights on genetic regulation of watermelon fruit quality development (Ren et al., 2014, 2018, 2020; Guo et al., 2015; Wang et al., 2017). However, most transcriptome studies have utilized the white-fleshed citron watermelon line PI 296341-FR for comparisons (Sandlin et al., 2012; Ren et al., 2014, 2018, 2020; Guo et al., 2015; Zhou et al., 2016; Wang et al., 2017; Zhang et al., 2017, 2020b; Anees et al., 2021). White-fleshed citron watermelon is non-sweet type because of accumulating low concentrations of sugars. Also, these genotypes show no changes in flesh color development during ripening (Ren et al., 2014, 2018; Guo et al., 2015; Anees et al., 2021) attributed to the low expression of genes involved in sugar and carotenoid metabolism (Guo et al., 2015).

There is limited comparative transcriptome analysis involving yellow and orange-fleshed citron watermelon genotypes. Compared to white-fleshed types, yellow and orange-fleshed types show extensive variations in flesh color development at various fruit developmental stages attributable to changes in carotenoid metabolism. The ripened fruit is slightly sweet- and- sour (TSS ∼4.0) (Ngwepe et al., 2021), probably attributable to high expression levels of genes involved in the biosynthesis of organic acids and sugars (Umer et al., 2020a). Also, the ripened citron watermelon fruit have a longer shelf life of up to 12 months under room temperature and up to 6 months in the open field (Personal observation). The extended shelf life of citron watermelon may be linked to low expression of ethylene and ABA biosynthesis genes leading to low accumulation of these phytohormones, and the high contents of cellulose, pectin and hemicellulose attributed to the high expression levels genes involved in cell-wall structural components (Guo et al., 2015; Zhou et al., 2016; Sun et al., 2020; Anees et al., 2021). These probably leads to the improved structural integrity of the rind and flesh, and low flesh degradation resulting in slow tissue breakdown increasing flesh firmness and extended shelf life (Sun et al., 2020; Anees et al., 2021). Therefore, comparative transcriptome analysis of gene expression involved in the regulation of fruit quality may reveal novel genes involved in watermelon fruit quality development. Therefore, we propose comparative transcriptome analysis as follows: (1) comparisons of yellow vs. orange-fleshed genotypes of *C. lanatus var. lanatus* and *C. lanatus var. citroides*, (2) yellow vs. orange-fleshed genotypes of *C. lanatus var. citroides* and (3) white vs. yellow vs. orange-fleshed genotypes of *C. lanatus var. lanatus* and *C. lanatus var. citroides.* These may reveal new insights on genetic regulation of watermelon fruit ripening and lead to molecular breeding strategies to improve sugars, carotenoids, organic acids, amino acid contents, and flavor and aroma (i.e., VOCs) and shelf-life of watermelon. These will result in new cultivar and product development to serve the diverse markets of the crop.

## Perspective

Watermelon fruit and flesh quality are affected by the composition of phytochemical compounds, phytohormones, and fruit flesh firmness which are affected by genes, the growing environment and their interaction. These attributes determine fruit ripening, eating quality, and postharvest shelf-life. For accelerated cultivar and product development to serve the changing needs of end-users, genes associated with fruit quality can be targeted for mutagenesis using gene editing platforms. Comparative transcriptome studies using yellow and orange-fleshed citron watermelon genetic resources is recommended to improve our understanding of genes involved in ripening and fruit quality development of watermelon fruit. Studies on QTL mapping and molecular marker development linked to organic acids, VOCs and fruit flesh structural components is proposed for marker-assisted breeding for accelerated product development, release and commercialization.

## Author Contributions

All authors listed have made a substantial, direct, and intellectual contribution to the work, and approved it for publication.

## Conflict of Interest

The authors declare that the research was conducted in the absence of any commercial or financial relationships that could be construed as a potential conflict of interest.

## Publisher’s Note

All claims expressed in this article are solely those of the authors and do not necessarily represent those of their affiliated organizations, or those of the publisher, the editors and the reviewers. Any product that may be evaluated in this article, or claim that may be made by its manufacturer, is not guaranteed or endorsed by the publisher.
